# Targeting senescence improves angiogenic potential of adipose-derived mesenchymal stem cells in patients with preeclampsia

**DOI:** 10.1186/s13293-019-0263-5

**Published:** 2019-09-14

**Authors:** Sonja Suvakov, Hajrunisa Cubro, Wendy M. White, Yvonne S. Butler Tobah, Tracey L. Weissgerber, Kyra L. Jordan, Xiang Y. Zhu, John R. Woollard, Fouad T. Chebib, Natasa M. Milic, Joseph P. Grande, Ming Xu, Tamara Tchkonia, James L. Kirkland, Lilach O. Lerman, Vesna D. Garovic

**Affiliations:** 1 Division of Nephrology and Hypertension, Mayo Clinic, 200 First Street SW, Rochester, MN 55905 USA; 2 Division of Maternal-Fetal Medicine, Department of Obstetrics and Gynecology, Mayo Clinic, Rochester, MN USA; 3 Department of Medical Statistics and Informatics, Medical Faculty, University of Belgrade, Belgrade, Serbia; 4 Department of Laboratory Medicine and Pathology, Mayo Clinic, Rochester, MN USA; 5 Kogod Center of Aging, Mayo Clinic, Rochester, MN USA; 6 Division of General Internal Medicine, Mayo Clinic, Rochester, MN USA

**Keywords:** Preeclampsia, Mesenchymal stem cells, Senescence, Angiogenesis, Senolytics, Dasatinib

## Abstract

**Background:**

Preeclampsia is a pregnancy-specific hypertensive disorder characterized by impaired angiogenesis. We postulate that senescence of mesenchymal stem cells (MSC), multipotent cells with pro-angiogenic activities, is one of the mechanisms by which systemic inflammation exerts inhibitory effects on angiogenesis in preeclampsia.

**Methods:**

MSC were isolated from abdominal fat tissue explants removed during medically indicated C-sections from women with preeclampsia (PE-MSC, *n* = 10) and those with normotensive pregnancies (NP-MSC, *n* = 12). Sections of the frozen subcutaneous adipose tissue were assessed for inflammation by staining for tumor necrosis factor (TNF)-alpha and monocyte chemoattractant protein (MCP)-1. Viability, proliferation, and migration were compared between PE-MSC vs. NP-MSC. Apoptosis and angiogenesis were assayed before and after treatment with a senolytic agent (1 μM dasatinib) using the IncuCyte S3 Live-Cell Analysis System. Similarly, staining for senescence-associated beta galactosidase (SABG) and qPCR for gene expression of senescence markers, p16 and p21, as well as senescence-associated secretory phenotype (SASP) components, IL-6, IL-8, MCP-1, and PAI-1, were studied before and after treatment with dasatinib and compared between PE and NP.

**Results:**

After in vitro exposure to TNF-alpha, MSC demonstrated upregulation of SASP components, including interleukins-6 and -8 and MCP-1. Staining of the subcutaneous adipose tissue sections revealed a greater inflammatory response in preeclampsia, based on the higher levels of both TNF-alpha and MCP-1 compared to normotensive pregnancies (*p* < 0.001 and 0.024, respectively). MSC isolated from PE demonstrated a lower percentage of live MSC cells (*p* = 0.012), lower proliferation (*p* = 0.005), and higher migration (*p* = 0.023). At baseline, PE-MSC demonstrated a senescent phenotype, reflected by more abundant staining for SABG (*p* < 0.001), upregulation of senescence markers and SASP components, as well as lower angiogenic potential (*p* < 0.001), compared to NP-MSC. Treatment with dasatinib increased significantly the number of apoptotic PE-MSC compared to NP-MSC (0.011 vs. 0.093) and decreased the gene expression of p16 and six SASP components. The mechanistic link between senescence and impaired angiogenesis in PE was confirmed by improved angiogenic potential of PE-MSC (*p* < 0.001) after dasatinib treatment.

**Conclusions:**

Our data suggest that MSC senescence exerts inhibitory effects on angiogenesis in preeclampsia. Senolytic agents may offer the opportunity for mechanism-based therapies.

## Background

Preeclampsia is a pregnancy-specific hypertensive disorder that is one of the leading causes of maternal and fetal morbidity and mortality [1]. This multi-system disease is commonly accompanied by proteinuria, occurs after 20 weeks of gestation, and affects approximately 5% of all pregnancies [1]. The etiology and pathogenesis of preeclampsia remain elusive, resulting in a failure to develop specific treatment strategies. Delivery remains the only therapy and commonly results in prematurity. It has been widely accepted that endothelial dysfunction and impaired angiogenesis play major roles in the development of preeclampsia. However, mechanisms underpinning the abnormal angiogenesis remain poorly understood.

Normal pregnancy is characterized by vigorous angiogenesis and maternal immune tolerance to the fetus due to suppressed Th1 (the cellular immune response potentially detrimental to the fetus) and enhanced Th2—humoral immunity—responses (“Th2 polarization”). Mesenchymal stem cells (MSC) contribute to the state of Th2 polarization and exhibit pro-angiogenic [2–4] and anti-inflammatory effects through the downregulation of tumor necrosis factor (TNF)-alpha and stimulation of interleukin (IL)-10 [5]. The natural corollary is that MSC dysregulation may contribute to abnormal angiogenesis and a systemic inflammatory state, thus leading to preeclampsia. However, MSC function in preeclampsia, with respect to systemic effects and angiogenic potential, has not yet been elucidated.

In this study, we postulated that impaired function and viability of MSC contribute to the anti-angiogenic and pro-inflammatory states in preeclampsia. We further posited that MSC dysfunction could be mechanistically related to cellular senescence, an irreversible cell-cycle arrest mechanism [6] that is characterized by apoptosis resistance and which is associated with a pro-inflammatory phenotype. As an initial response to insult, senescence may be advantageous by promoting tissue repair and regeneration and by guarding against unrestricted growth of damaged cells. As such, senescence plays an important physiological role in embryonic development and tissue healing. However, evoked in response to stress, the irreversible proliferative arrest leads to systematic metabolic and functional decline. The senescent cell state is mediated by p16 and p21 and senescence-associated tissue injury is caused, in part, by release of pro-inflammatory markers, commonly referred to as the senescence-associated secretory phenotype (SASP) [6]. Aberrant placental aging and increased placental senescence have been demonstrated in preeclamptic placentas [7]. Several SASP components have been shown to be elevated in women with preeclamptic compared to normotensive pregnancies, including the major SASP components, such as IL-6 [8], IL-8 [9], plasminogen activator inhibitor-1 (PAI-1) [10], and monocyte chemotactic protein-1 (MCP-1) [11]. However, neither the role of MSC, in general, nor their senescence, in particular, has been studied in the context of impaired angiogenesis in preeclampsia. One feasible approach to obtain MSC at the time of delivery is to collect them from adipose tissue during C-section. Of note, the characteristics of MSC residing in different organs are similar [12], suggesting that the functional status of adipose tissue-MSC is representative of diverse MSC in a given individual.

To discern the role of MSC dysregulation in preeclampsia, we compared the viability and function of MSC harvested from adipose tissue from women with preeclampsia vs. women with normotensive pregnancies at the time of delivery. We further compared senescent cell burden, markers of MSC senescence, the SASP, and MSC angiogenic potential between the groups, both before and after treatment with dasatinib, a senolytic agent. As a class, senolytic agents target survival pathways in senescent cells, causing apoptosis without significant effects on quiescent or proliferating cells [13]. Based on our previous observations that targeting senescent cells may prevent or delay tissue dysfunction [14, 15], we postulated that senolytic treatment may decrease the senescent MSC burden in preeclampsia and improve their angiogenic potential.

## Methods

### Participants

Women with preeclampsia and normotensive pregnant women were recruited from the Mayo Clinic Family Birth Center. All women delivered by clinically indicated Cesarean section. This study was approved by the Mayo Clinic Institutional Review Board (IRB), protocol # 2105-05, and all participants provided written informed consent prior to participating. Each participant’s medical record was reviewed to confirm the pregnancy diagnosis and obtain information on demographic characteristics and pregnancy outcomes. The diagnosis of preeclampsia was based on the presence of the following criteria [16]: hypertension after 20 weeks gestation, defined as (a) blood pressure ≥ 140/90 mmHg; (b) proteinuria, defined as ≥ 300 mg of protein in a 24-h urine specimen, and/or protein/creatinine (Cr) ratio of 0.3, and/or 1+ (30 mg/L) dipstick urinalysis in the absence of urinary tract infection. In the absence of proteinuria, the diagnosis of preeclampsia was confirmed if any of the following applied: (i) laboratory abnormalities, including thrombocytopenia < 100,000/μL, serum Cr > 1.1 mg/dL or its doubling, and elevated liver function tests, AST and ALT (> 2× ULN), were present; or (ii) the presence of pulmonary edema or cerebral or visual symptoms [16]. Women were classified as having a normotensive pregnancy if they showed no signs of hypertension throughout gestation.

### MSC isolation from adipose tissue

Abdominal fat tissue (3–5 g) obtained during C-section was cultured at 37 °C/5% CO_2_ in Advanced MEM media supplemented with 5% platelet lysate (PLTmax, Mill Creek Life Sciences, Rochester, MN), which provides a robust growth medium [17, 18]. The third passage of cells was used for phenotype/function analysis [19–24]. MSC were positive for CD90, CD44, and CD105 and negative for CD34, CD31, and CD45 by Flow Cytometry (FlowSight,™ Amnis, Seattle, WA), and were able to transdifferentiate into adipocytes, chondrocytes, and osteocytes. For studies in non-pregnant subjects, MSC were isolated from three healthy kidney donors at time of kidney donation Mayo Clinic (IRB) for Human Research (IRB#11-009182).

### In vitro effects of TNF-alpha on MSC

MSC isolated from healthy kidney donors were treated with vehicle or 20 ng/mL TNF-alpha for 24 h. After incubation, MSC were washed and RNA isolated, and gene expression of inflammatory cytokines was measured using q-PCR, as described under the “Analysis of gene expression by qPCR” section.

### MSC function

MSC function was assessed by proliferative and migrating capabilities, as we have described previously [19–21, 24–27]. In brief, MSC migratory function was tested using a QCM^TM^ Chemotaxis Cell Migration kit (ECM508, EMD Millipore) [26] and proliferative activity by MTS (Promega). Proliferation and migration were measured at 490 and 560 nm, respectively, using the SynergyMX spectrophotometer (BioTek Instruments, Inc., Winooski, VT), and expressed in optical density (OD) units.

### Cell viability

Cell viability was measured using Flow Cytometry for Annexin V, as previously described [28].

### Immunohistochemistry

Five-micrometer-thick sections of frozen subcutaneous adipose tissue were processed following standard protocols. Inflammation was assessed by staining for TNF-alpha (1:100, Santa Cruz Biotechnology) and monocyte chemoattractant protein (MCP)-1 (1:100, Abcam); oxidative stress was evaluated by the in situ production of superoxide anion and detected by fluorescence microscopy using dihydroethidium (DHE). Image analysis utilized a computer-aided image-analysis program (AxioVision Carl Zeiss Micro Imaging, Thornwood, NY). Results were expressed as percent of the field-of-view staining (average of 4–6 fields).

### Apoptosis assay for MSC

Apoptosis was assessed by Annexin V reagent (Essen Bioscience) using the IncuCyte S3 Live-Cell Analysis System (Essen Bioscience).

### Angiogenesis assay

The angiogenic potential of MSC was assessed using human umbilical vein endothelial cells (HUVEC) angiogenesis assay. NP-MSC and PE-MSC were transferred to a 96-well plate (Corning Incorporated, USA) at 4000 cells per well where they were co-cultured with previously seeded GFP-expressing HUVEC (IncuCyte CytoLight Green HUVEC Cells) and human fibroblasts (IncuCyte NHDF Cells) as instructed in the manufacturer’s kit. The plate was placed in the IncuCyte S3 Live-Cell Analysis System where real-time images were captured every 3 h. Angiogenesis was assessed as the total network length (mm/mm^2^) using IncuCyte S3 Software (Essen Bioscience) and compared between groups.

### Senescence-associated beta galactosidase (SABG) staining

For SABG staining, 50,000 MSC were seeded in a 12-well plate and left until they had reached 70–80% confluency. Cells were fixed in beta galactosidase fixation solution for 10 min and washed twice with PBS. Subsequently, the cells were stained overnight using SABG reagent (Cell Signaling Technology) according to the manufacturer’s directions. Nuclei for DAPI imaging were stained using Hoechst reagent. Image analysis utilized a computer-aided image-analysis program (AxioVision Carl Zeiss Micro Imaging, Thornwood, NY). Results are shown presented as percent of the stained cells in field-of-view (average 8–10 fields).

### Analysis of gene expression by qPCR

MSC were collected and stored at − 80 °C until further use. RNA was isolated by QIAzol Lysis Reagent and RNeasy Mini Columns (QIAGEN, Valencia, CA) following the manufacturer’s instructions. RNA concentration and 230/260 absorbance ratios were checked using a NanoDrop spectrophotometer (Thermo Scientific, Wilmington, DE). cDNA was synthesized and qPCR was performed using TaqMan™ Fast Advanced Master Mix on Biorad CXF96 Platform in a 10 μL volume using the following thermal protocol: 50 °C for 2 min, 45 cycles of 95 °C for 20 s, and 60 °C for 30 s. Gene expression was normalized to TATA-box-binding protein (TbP). The following primers were purchased from Applied Biosciences: total p16 (catalog number: Hs00923894), p21 (catalog number: Hs00355782), IL-6 (catalog number: Hs00174131), IL-8 (catalog number: Hs00174103), MCP-1 (catalog number: Hs00234140), PAI-1 (catalog number: Hs01126607), and PAI-2 (catalog number: Hs00299953).

### Treatment with dasatinib

The initial dose response experiments were performed to determine the optimal dasatinib concentration using the apoptotic assay and IncuCyte S3 Live-Cell Analysis System (Essen Bioscience). Approximately 1 × 10^6^ MSC (PE and NP) in passage #4 were treated with the senolytic drug, dasatinib, at concentrations of 1, 2, 5, and 10 μM (dissolved in 0.1% DMSO) for 24 h. Three groups were analyzed: (1) cells incubated in media, (2) vehicle—cells treated with 0.1% DMSO, and (3) cells treated with dasatinib. The MSC were seeded at 5000 cells/well in 96-well plates (Advanced MEM with 10% FBS) and treated with dasatinib at increasing concentrations. Annexin V, added at the beginning of the treatment, labeled apoptotic cells yielding red fluorescence. The plate was scanned at a magnification of 10× and degree of fluorescence and images were assessed and taken in real time from the beginning of the treatment up to 24 h post treatment. Using IncuCyte S3 Software, we generated a red object count per well at each time point. Ratios of apoptotic cells in the dasatinib-treated, vehicle-treated, and media groups were compared and used to determine that the optimal concentration of dasatinib was 1 μM (see the “Results” section) for studying effects of this senolytic agent on the burden of senescent PE-MSC (SABG, SASP, p16, and p21) and their functional angiogenic potential.

### Statistical analysis

Descriptive statistics of demographic and clinical characteristics are reported as mean ± SD, median and interquartile range (IQR), or number and percentage, as appropriate. Group differences between women with normotensive pregnancy and those with preeclampsia were determined by the Student *t* test or/and ANOVA for repeated measurements. Correlations were analyzed using Pearson’s correlation coefficient. Graphics for plotting individual level data were created using an interactive graph tool [29] and GraphPad Prism 8 (RRID: SCR_002798). Correlations among various parameters were analyzed using Pearson’s correlation or the Spearman’s correlation coefficient. All data analyses were performed using SPSS statistical software, version 25 (IBM SPSS, Chicago, IL, RRID: SCR_002865), with significance determined on the basis of *α* = 0.05.

## Results

### Clinical characteristics of participants

Maternal age did not differ between women with preeclampsia and those with normotensive pregnancy. Women with preeclampsia delivered earlier in pregnancy compared to women with normotensive pregnancies and, as expected, had higher systolic and diastolic blood pressures (Table 1). Gestational diabetes and twin pregnancy, known risk factors for preeclampsia, were each documented in 20% of the PE pregnancies. Six of 10 preeclamptic pregnancies had clinical evidence of co-existing HELLP (*h*emolysis, *e*levated *l*iver enzymes, *l*ow *p*latelet count) syndrome.

**Table 1:** Baseline characteristics of women with normotensive versus preeclamptic pregnancies

Variable	Normotensive (*n* = 12)	Preeclampsia (*n* = 10)	*p*
Age at delivery, years, mean ± SD	32.3 ± 5.5	29.5 ± 5.7	0.253
Gestational age, weeks, mean ± SD	38.6 ± 0.8	35.3 ± 4.0	0.011
Systolic blood pressure, mmHg, mean ± SD	111.6 ± 11.5	152.5 ± 3.1	< 0.001
Diastolic blood pressure, mmHg, mean ± SD	64.4 ± 11.0	100.8 ± 12.4	< 0.001
Proteinuria, mg/24 h, med (IQR)	N/A	635 (252–1917)	
GDM, *n*	0	2	
Twins, *n*	1	2	
BMI	34.0 ± 8.7	32.3 ± 7.1	0.617

Results are expressed mean ± standard deviation

HELLP syndrome

N/A for Normotensive and 6 for Preeclampsia

*GDM* gestational diabetes mellitus, *BMI* body mass index, *HELLP h*emolysis, *e*levated *l*iver enzymes, *l*ow *p*latelet count; *N/A*, not applicable

### In vitro effects of TNF-alpha on MSC from healthy, non-pregnant subjects

**Fig. 1:**
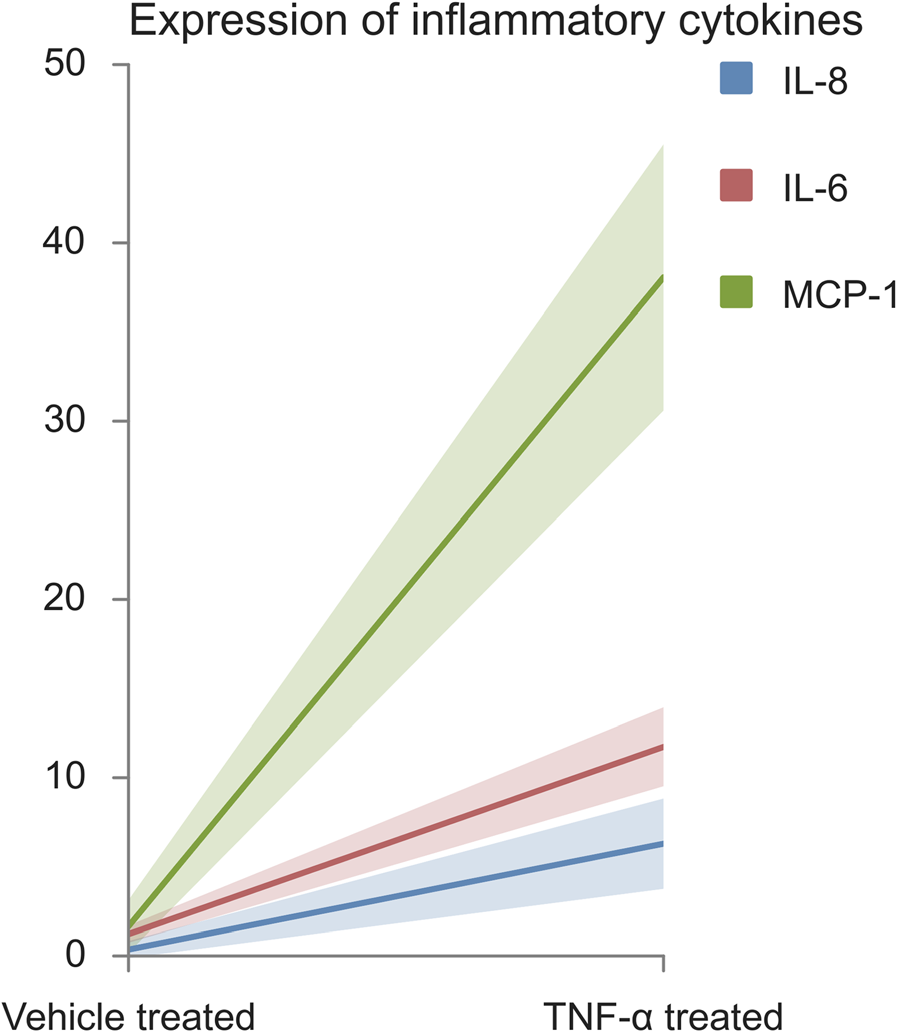
Expression of inflammatory cytokines in non-pregnant woman MSC treated for 24 h with TNF-alpha. All three markers tested were significantly elevated after treatment with TNF-alpha vs. vehicle (presented as mean ± SD): IL-6 (in red), 11.73 ± 2.20 vs. 1.22 ± 0.47 (*p* = 0.009); IL-8 (in blue), 6.29 ± 2.53 vs. 0.36 ± 0.47 (*p* = 0.38); MCP-1 (in green), 38.07 ± 7.46 vs. 1.65 ± 1.46 (*p* = 0.010), respectively

Abdominal fat tissue was obtained from three healthy kidney donors aged 39 ± 3.3 years with a body mass index of 26.6 ± 0.9 (mean ± SEM) at time of kidney donation. MSC were isolated and characterized as described in the “Methods” section. After co-incubation with vehicle or TNF-alpha (20 ng/mL) for 24 h, expression of the inflammatory cytokines, interleukin (IL)-6, IL-8, and MCP-1, were significantly elevated in the TNF-alpha compared to the vehicle-treated MSC (Fig. 1).

### Adipose tissue immunocytochemistry in pregnancy

**Fig. 2:**
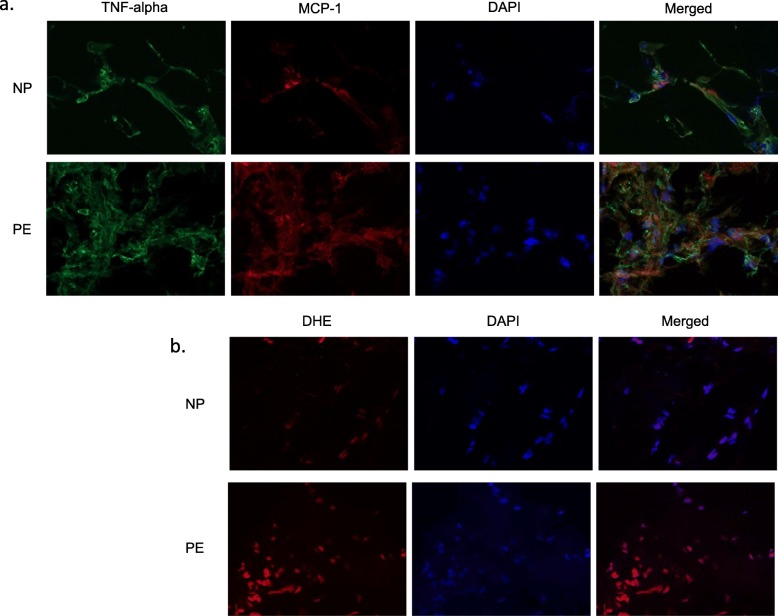
Fat tissue staining for markers of inflammation and oxidative stress in normotensive pregnant (NP) (upper rows) and preeclamptic (PE) women (lower rows). TNF-alpha and MCP-1 were upregulated in PE. Representative images for TNF-alpha, MCP-1, and DAPI (4,6-Diamidino-2-phenylindole, dihydrochloride) nuclear (nucleus) staining, as well as merged TNF-alpha and MCP-1 (**a**). DHE (dihydroethidium) staining tended to be increased in PE compared to NP. Representative images for DHE and DAPI, as well as merged DHE and DAPI (**b**)

Adipose tissue staining revealed higher expression of TNF-alpha and MCP-1 in preeclampsia compared to normotensive pregnancy (Table 2, Fig. 2a; *p* < 0.001 and *p* = 0.024, respectively), indicating increased fat inflammation. A trend towards higher DHE staining (*p* = 0.084) suggested a tendency for increased oxidative stress in the abdominal fat from women with preeclampsia (Fig. 2b).

**Table 2:** MSC viability, function, and immunocytochemistry of fat tissue in women with normotensive vs. preeclamptic pregnancies

Variable	Normotensive/preeclamptic samples	Normotensive	Preeclampsia	*p*
Live cells, %	12/10	92.47 (91.53–93.41)	88.50 (85.12–91.86)	0.012
Dead cells, %	12/10	4.21 (3.57–4.86)	7.16 (4.41–9.91)	0.019
Proliferation (OD)^x^	10/7	0.80 (0.62–0.99)	0.44 (0.32–0.57)	0.005
Migration (OD)^x^	10/7	1.30 (0.99–1.61)	1.88 (1.42–2.33)	0.023
DHE^†^	12/9	0.287 (0.241–0.334)	0.352 (0.284–0.419)	0.084
TNF-alpha^†^	12/9	0.090 (0.058–0.122)	0.284 (0.210–0.358)	< 0.001
MCP-1^†^	12/9	0.058 (0.041–0.075)	0.085 (0.069–0.101)	0.024

Results are expressed as mean (95% CI)

*DHE* dihydroethidium, *TNF-alpha* tumor necrosis factor-alpha, *MCP-1* monocyte chemoattractant protein-1, *OD* optical density units

^x^Proliferation and migration measured at 490 and 560 nm wavelengths, respectively

^†^Expressed as the percent of the field-of-view staining (average of 4–6 fields)

### MSC viability, proliferation, and migration

**Fig. 3:**
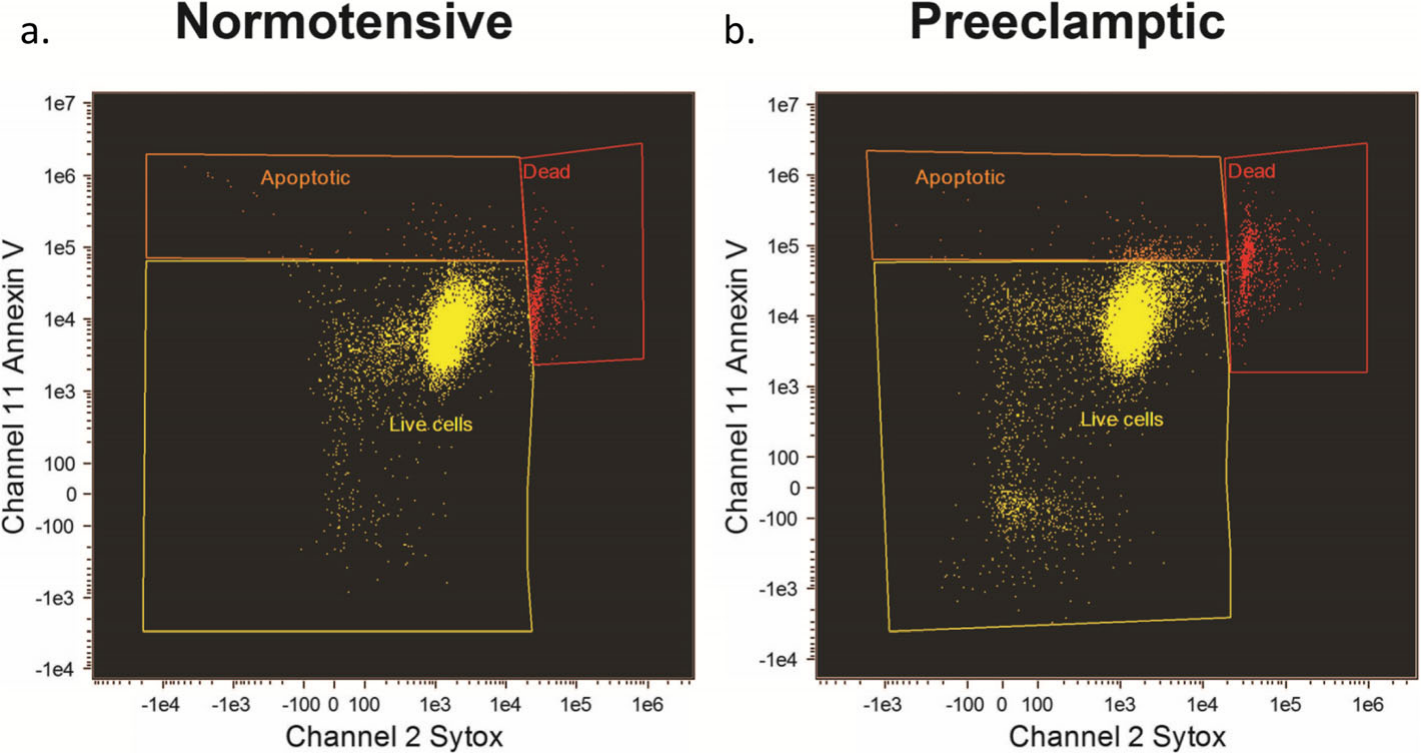
Representative flow cytometry scatterplots of MSC viability. MSC viability tested using Annexin V (channel 11) and Sytox (Channel 2) shows decreased MSC viability in preeclamptic (85%) vs. normotensive (94%) pregnancies (*p* = 0.01). This is a representative image where yellow panel represents live cells, red panel represents dead cells, and orange panel represents apoptotic cells

Cell viability was reduced in preeclampsia (Table 2). Women with preeclampsia had a lower percentage of live MSC cells (*p* = 0.012) and a higher percentage of dead cells (*p* = 0.019) than normotensive pregnant women (Fig. 3a, b, Table 2). Significantly lower proliferation (*p* = 0.005) was observed in PE-MSC compared to NP-MSC. In contrast, PE-MSC demonstrated higher migration (*p* = 0.023). The average proliferation value was positively correlated with the percentage of live cells (*r* = 0.641, *p* = 0.006) and correlated negatively with the percentage of dead cells (*r* = − 0.659, *p* = 0.004).

### Higher senescent cell burden, upregulation of senescence markers, and SASP components are present in preeclamptic compared to normotensive MSC

**Fig. 4:**
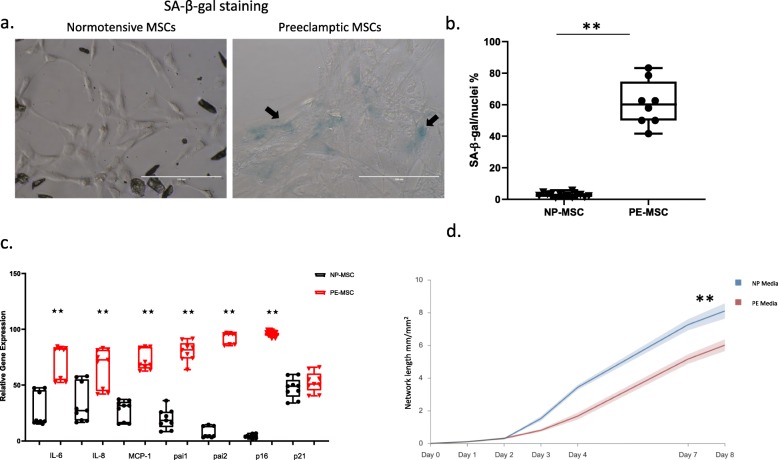
Senescent cell burden is higher in PE-MSC compared to NP-MSC. SABG staining revealed a higher number of stained cells (marked with black arrows) in PE-MSC compared to their normotensive counterparts (**a**). Data presented as mean values of SABG-stained MSC with min-max (**b**). Expression of p16, but not p21, was significantly increased. All SASP genes were significantly more highly expressed in PE-MSC compared to NP-MSC. Data are shown as box-plots (min-max) with all individual values (**c**). PE-MSC and NP-MSC were co-cultured with GFP expressing HUVEC for 8 days in total, and total network length was measured continuously every 3 h. Significantly lower angiogenic potential was registered for HUVEC co-cultured with PE-MSC, compared to NP-MSC (*F* = 13.965; df = 8, *p* < 0.001) (**d**)

Isolated PE-MSC and NP-MSC, from preeclamptic and normotensive pregnant patients, respectively, were stained for SABG. The number of senescent cells, as determined by SABG staining, was significantly higher in PE-MSC, with approximately 60.8 ± 14.3% of counted cells being senescent, compared to 2.8 ± 1.3% of the NP-MSC (*p* < 0.001) (Fig. 4a, b). Expression of senescence markers and SASP-related genes was assessed in both groups. PE-MSC had significantly higher expression of the senescence marker, the p16 gene (*p* < 0.001), compared to NP-MSC, but not p21 (*p* = 0.999). All SASP-related genes demonstrated significantly higher expression in PE-MSC compared to NP-MSC (IL-6 *p* < 0.001, IL-8 *p* = 0.040, MCP-1 *p* < 0.001, PAI-1 *p* < 0.001, PAI-2 *p* < 0.001) (Fig. 4c).

### Preeclamptic MSC exhibit low angiogenic potential

In order to examine and compare the angiogenic potential of MSC, the total network length (mm/mm^2^) of endothelial cells developed during co-culture with MSC was measured. We showed that PE-MSC exhibit lower angiogenic potential compared to their normotensive counterparts (*p* < 0.001) when incubated in the medium (Fig. 4d). Monitoring the network length formation of endothelial cells was continuous for 8 days, with the observation that the angiogenic potential of PE-MSC was significantly lower compared to the NP-MSC (*F* = 13.965; df = 8, *p* < 0.001).

### Apoptotic effects of senolytic agent (dasatinib) on MSC

**Fig. 5:**
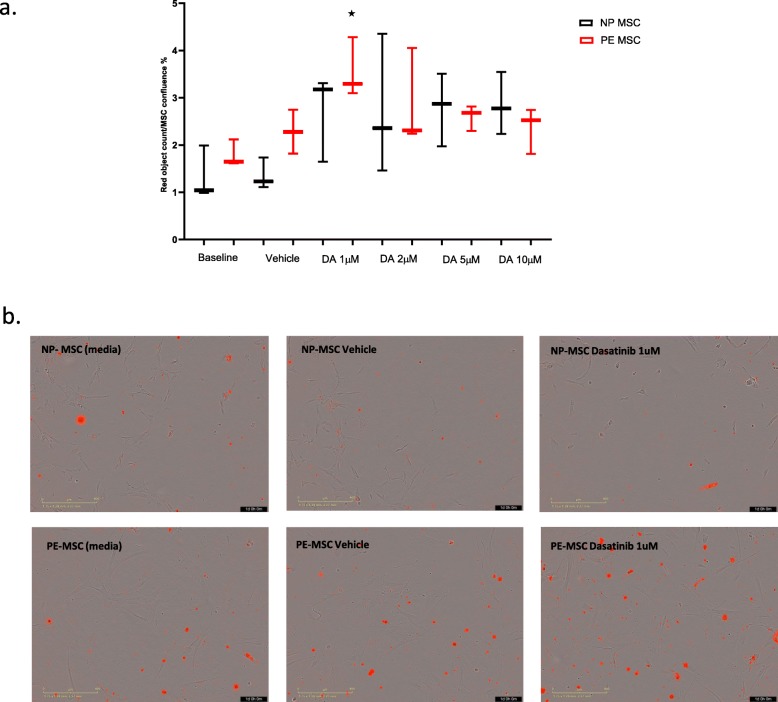
Apoptotic effects of the senolytic agent, dasatinib, on MSC. Dose-response experiments showed that PE-MSCs are sensitive to a lower concentration (1 μM) of dasatinib, while the apoptotic effect of the drug is lower at higher concentrations of the drug (**a**). Treatment with 1 μM dasatinib revealed significant apoptotic effects in PE-MSC (*p* = 0.0117) compared to the non-treated cells, but not in NP-MSC (*p* = 0.0934). Representative image shows apoptotic cells stained red for PE-MSC and NP-MSC in all three conditions (medium, vehicle, treatment) (*n* = 3) (**b**)

To determine the optimal senolytic drug concentration, both PE and NP-MSC were treated with four different concentrations of dasatinib: 1 μM, 2 μM, 5 μM, and 10 μM. Accumulation of apoptotic bodies (red object count) was assayed after 24-h treatment in Incucyte. PE-MSC were prone to apoptosis when treated with lower concentrations of dasatinib. Increasing concentrations of the drug did not result in a further increase in apoptosis. At the same time, NP-MSC were more sensitive to the apoptotic effects of the drug when treated with higher concentrations of dasatinib (Fig. 5a). Notably, dasatinib at a concentration of 1 μM induced significant apoptosis in PE-MSC (*p* = 0.0117), but not in NP-MSC (*p* = 0.0934), compared to the cells not treated with the drug (Fig. 5b). Based on this, 24-h treatment with 1 μM dasatinib was used for further experiments in this study.

### Treatment with dasatinib improves angiogenic potential of PE-MSC

**Fig. 6:**
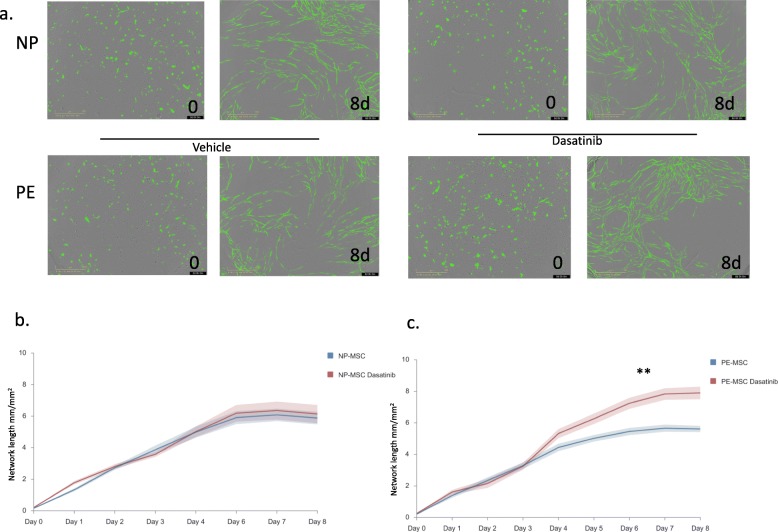
Angiogenic potential of dasatinib-treated PE-MSC was improved after the treatment. Representative images showing the total network length formed at day 0 and day 8 after senolytic agent treatment of PE-MSC and NP-MSC (**a**). While no significant difference in angiogenic potential of NP-MSC (*n* = 9) was observed after the treatment (*F* = 0.406; df = 8; *p* = 0.916) (**b**), NP-MSC co-cultured HUVEC showed significant improvement in angiogenesis (*F* = 22.436; df = 8; *p* < 0.001) (**c**)

To test whether treatment with dasatinib improves the angiogenic potential of PE-MSC, we treated the cells with this senolytic drug as described above. After the treatment, cells were co-cultured with green-labeled HUVEC and the total network length development was monitored for a total of 8 days (Fig. 6a). There was no significant change in the total network length of HUVECs between treated and untreated NP-MSC (Fig. 6b, *F* = 0.406; df = 8; *p* = 0.916). During the first 4 days, there was no significant difference in the total network length of endothelial cells co-cultured with PE-MSC. However, beginning on day 5, PE-MSC treated with dasatinib had significantly improved angiogenic potential compared to the non-treated PE mesenchymal cells (Fig. 6c, *F* = 22.436; df = 8; *p* < 0.001).

### Treatment with dasatinib decreases senescent cell burden and expression of senescence markers and SASP components in PE-MSC

**Fig. 7:**
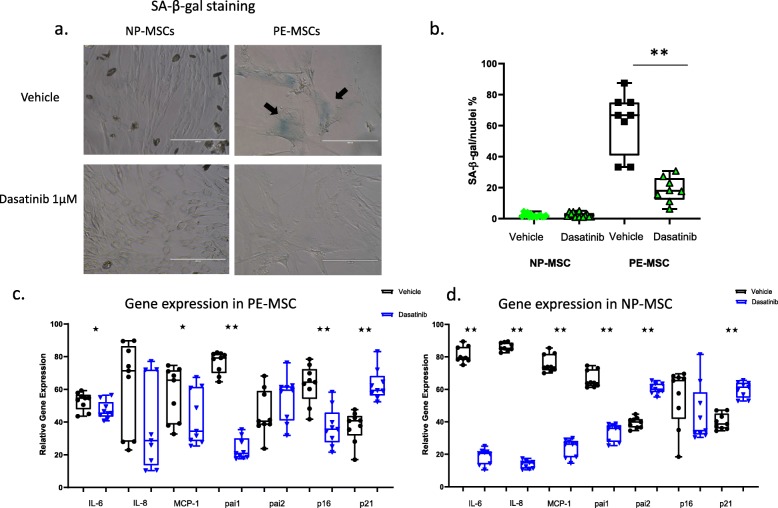
Treatment with dasatinib cleared senescent cells from PE-MSC and affected senescence-related gene expression. Representative images from SABG staining show abundant senescent cells in PE-MSC (marked with black arrows), but not in NP-MSC, in vehicle (**a**). Dasatinib treatment completely removed senescent cells from PE-MSC (*p* < 0.001) (**b**). PE-MSC had a significant decrease in expression of p16 (*p* = 0.025) and PAI-1 (*p* < 0.001), IL-6 (*p* = 0. 0487), and MCP-1 (*p* = 0.040), while IL-8 (*p* = 0.136) was modestly decreased after treatment. Significantly increased expression of the p21 and PAI-2 genes was observed after the treatment (**c**). Relative expression of the senescence marker gene, p16, in NP-MSC after treatment with Dasatinib remained unchanged (*p* = 0.136). Apart from p21 and PAI-2 whose expression increased in a manner similar to PE-MSC, the relative gene expression of the other tested genes was decreased in NP-MSC after the treatment with dasatinib (*p* < 0.001) (**d**)

To demonstrate that dasatinib can remove cells with the senescent phenotype, both PE-MSC and NP-MSC were stained for SABG before and after treatment with dasatinib. Treatment with dasatinib completely removed SABG-stained cells from the culture of PE-MSC (non-treated PE-MSC = 62.5 ± 19.5% vs. treated PE-MSC = 18.7 ± 8.1%, *p* < 0.0001) (mean ± SD) (Fig. 7a, b). No difference in SABG staining in NP-MSC was observed (*p* = 0.642). In addition, dasatinib reduced the expression of senescence and SASP markers in both PE-MSC and NP-MSC. Thus, after senescent cell clearance, PE-MSC had significant decreases in the expression of p16 (*p* < 0.001), PAI-1 (*p* < 0.001), IL-6 (*p* = 0.0487), and MCP-1 (*p* = 0.040), while IL-8 (*p* = 0.136) showed a modest decrease in expression after treatment. On the other hand, the expression of p21 significantly increased (*p* < 0.001), followed by an increase in PAI-2 gene expression (*p* < 0.001) after treatment (Fig. 7c). Expression of the senescence marker, p16, in NP-MSC after treatment with dasatinib remained unchanged (*p* = 0.136). Relative gene expression of IL-6, IL-8, MCP-1, and PAI-1 was significantly decreased in NP-MSC after treatment with dasatinib (*p* < 0.001), while p21 and PAI-2 gene expression increased (Fig. 7d).

## Discussion

In the present study, we report several novel findings regarding the role of MSC in preeclampsia. First, our results demonstrate that viability and function of MSC harvested from adipose tissue at the time of delivery are impaired in preeclamptic compared to normotensive pregnancies. In contrast, the migration capacity of MSC was increased, possibly secondary to MCP-1 upregulation—a potent trophic factor for MSC—in neighboring fat. Second, we show that a pro-inflammatory adipose tissue *milieu*, as demonstrated by upregulation of TNF-alpha, is associated with upregulation of SASP components in PE-MSC compared to NP-MSC. The mechanistic link between inflammation and upregulation of SASP components was confirmed by experiments showing the upregulation of IL-6, IL-8, and MCP-1 in control MSC after exposure to TNF-alpha. Third, our results indicate decreased pro-angiogenic potential of PE-MSC and, fourth, provide evidence that this is due, at least in part, to their senescence, as treatment with dasatinib both decreases senescent MSC burden and results in improved MSC angiogenic potential. Taken together, our data suggest that the pro-inflammatory *milieu* of the abdominal tissue—where MSC reside—is associated with MSC senescence and both a decrease in MSC-mediated angiogenic effects and an increase in SASP components, the latter further contributing to the vicious cycle of inflammation → senescence → anti-angiogenesis. By implicating MSC senescence in the pro-inflammatory and anti-angiogenic mechanisms of preeclampsia, our study opens new avenues for preeclampsia treatment, such as autologous stem cell transplantation. If MSC function and viability appear suboptimal due to senescence, pre-intervention testing and pre-conditioning with senolytic agents may be considered. The process of senescence is critical for embryogenesis and, therefore senolytics are contraindicated during pregnancy. However, their therapeutic use in non-pregnant women could be considered for prevention of preeclampsia in patients with previous unsuccessful pregnancies due to preeclampsia and its complications. This approach would be facilitated by the mechanism of action of senolytics: these agents are effective when administered intermittently, and a single dose (or a short duration of treatment) would result in a decrease in senescent cell burden after the affected pregnancy and prior to planning the next one. Given their short elimination half-lives, the risk of adverse effects for future pregnancies would be minimal. In addition, senolytics could be considered during the post-reproductive years in women with a history of preeclampsia, who may experience persistent increase in senescent cell burden, potentially leading to increased risks for metabolic syndrome, an accelerated aging-like state, or multimorbidity. Of note, adverse effects of dasatinib are rare, appear only after prolonged administration of the drug, and are usually reversible after a dose interruption. We present here a proof of principal study showing a potential benefit of senescent cell clearance in preeclampsia. One alternative approach would be to use, in place of senolytics, senomorphic agents (those that that attenuate the SASP, such as metformin) [13], which are safe for use even in pregnancy. Finally, for these patients, the continued development of new senolytic drugs with less toxicity, which is underway, will be of particular importance.

Stem cells play fundamental roles in the repair and self-renewal of tissues throughout life. Tissue injury activates regenerative mechanisms that promote repair by recruiting local resident stem cells, bone marrow-derived hematopoietic progenitor/stem cells, or MSC. MSC are multipotent cells that have been identified in almost all tissues, including kidney and placenta [5]. MSC have a potent modulatory effect and may contribute to the state of Th2 polarization and immune tolerance in pregnancy, by either a direct inhibitory effect on the proliferation of Th1 cells or by shifting a Th1 to a Th2 phenotype. MSC also exhibit pro-angiogenic [2–4] and anti-inflammatory effects through downregulation of TNF-alpha and stimulation of IL-10 [5]. In pregnancy, MSC can traffic through the placenta, in a process triggered by fetal VEGF, and may be responsible for fetal microchimerism in normal pregnancy [30]. In addition, placental MSC reside in a peri-vascular niche in the developing placenta [31], with emerging evidence suggesting that they play an important role in placental development by contributing to vasculogenesis and angiogenesis. A study of the differential expression of microRNAs in decidua-derived MSC from severe preeclampsia and normal pregnancies indicated that angiogenesis, response to hypoxia, apoptosis, the TGF-beta receptor signaling pathway, cell migration, and immune response, were regulated by increased MSC microRNAs in patients with preeclampsia [32]. Taken together, MSC play an important role in the regulation of placentation in normal pregnancy. In turn, MSC dysregulation may contribute to the pathophysiology of preeclampsia. However, their function in preeclampsia with respect to their potency for attenuating inflammation and repairing vascular injuries has not yet been studied. Our study is the first to provide data supporting the notion that MSC are dysregulated in preeclampsia, and linking senescence of MSC to the anti-angiogenic state, one of the hallmarks of vascular injury in preeclampsia.

Data presented in this study need to be interpreted in the context of the current state of knowledge of preeclampsia pathophysiology and the widely accepted concept that maternal disease is caused by pro-inflammatory and anti-angiogenic mediators that are released by ischemic placenta. Previous studies have shown accelerated placental aging and increased placental senescence in preeclamptic placentas [7]. We postulate that, once established, increased placental senescent cell burden persists, with affected cells acquiring a SASP secretome consisting of circulating inflammatory cytokines and reactive oxygen species, ultimately leading to maternal vascular and tissue injury. Furthermore, we showed that senescence can spread from cell to cell [14], suggesting the possibility that senescent cells in the placenta could cause other cells to become senescent elsewhere in the mother, potentially establishing a reservoir of these cells. Therefore, the SASP secretome of placental origin may be one of the missing links between placental ischemia and maternal disease in preeclampsia. In future experiments, we will compare MSC in “placental” vs. “maternal” forms of preeclampsia, the former clinically characterized by early (≤ 34 weeks of gestation) and severe disease, placental changes of ischemia and infarction, and consequent intrauterine growth restriction; the latter described by preexisting maternal disease (hypertension, diabetes mellitus), late onset (> 34 weeks of gestation) with the absence of ischemic placental changes, and normal intrauterine fetal growth. We postulate that differential degrees of senescent cell burden may, indeed, contribute to the differences in clinical presentations between these disease subtypes.

Preeclampsia is commonly viewed as a vascular disease of pregnancy. Of note, reduction in capillary density in the skin has been reported in association with preeclampsia, both prior to the clinical onset of the disease [33] and at the time of diagnosis [34]. We postulated that MSC senescence and impaired angiogenesis may lead to sustained vascular injury and rarefaction. Indeed, the MSC from preeclamptic pregnancies showed increased staining for SABG, a marker of senescence. The mechanistic link between MSC dysfunction and senescence was studied using a senolytic agent as a means of rescuing functional phenotypes. Senolytic agents promote selective apoptosis in senescent, but not normal cells, by transiently disabling their pro-survival pathways [13, 35]. The resultant decrease in senescent cell number and SASP inhibition have been shown to delay or alleviate age- and disease-related adverse phenotypes, as well as to improve established vascular disease in aged and hypercholesterolemic mice [36, 37]. In the current study, the mechanistic link between MSC dysfunction and senescence was studied using dasatinib, a tyrosine kinase inhibitor that specifically targets senescent MSC. The following outcomes of dasatinib treatment of MSC support the role of MSC senescence in preeclampsia. First, senescent MSC burden decreased after treatment. Second, the number of apoptotic MSC in preeclampsia increased, as expected to occur with senolytic agents, which target survival pathways in senescent cells and cause their apoptosis. Third, the angiogenic potential of PE-MSC significantly improved after treatment. It is noteworthy that downregulation of SASP components occurred in both NP-MSC and PE-MSC. It has been widely accepted that even normal pregnancy is associated with systemic inflammation, which is further exaggerated in preeclamptic pregnancies [38]. Consequently, down-regulation of the SASP in both groups can be attributed to anti-inflammatory effects of dasatinib. However, the downregulation of the SASP was associated with downregulation of p16 in only PE-MSC, but not NP-MSC. Furthermore, only PE-MSC, but not NP-MSC, demonstrated improved angiogenic potential after treatment. Taken together with the results of SABG staining, these data indicate that improved PE-MSC angiogenic potential was achieved through a decrease in senescent cell burden.

A notable limitation of our study is its small sample size, which did not allow for the characterization of MSC viability and function across the spectrum of severity (mild vs. severe) and presentations (early vs. late) of preeclampsia; these will be addressed in ongoing studies in our laboratory. Also, only a single time point was studied. While the mechanistic link between inflammation and upregulation of SASP components was confirmed by experiments showing the upregulation of IL-6, IL-8, and MCP1 in control MSC after exposure to TNF-alpha, the observed differences between PE-MSC and NP-MSC could be secondary to hypertension and differences in gestational age. Although it would be reasonable to examine endometrial MSC in preeclampsia for purposes of studying placental physiology, we opted for abdominal fat MSC for several reasons. First, the characteristics of MSC residing in the different organs are similar [12], suggesting that, in a given subject, the functional status of diverse MSC is comparable. Second, if autologous stem cell transplant is to be considered as a potential therapy for preeclampsia, fat tissue is readily accessible and abundantly available. Characterization of adipose tissue-derived MSC is critical to advance toward this goal. Third, this approach would allow for longitudinal examination of MSC obtained at the time of delivery to those collected postpartum in future studies. Collection of endometrial MSC would be feasible only at the time of delivery, e.g., during C-section.

Despite these limitations, our study reveals novel insights involving MSC senescence in preeclampsia, which may open new venues for preeclampsia research and novel treatment strategies. While stem cell therapies to treat placental disorders may seem like a far-fetched concept [5], stem cells are explored for therapeutic use and have been found to be effective in a broad spectrum of disease entities [39]. This study implies, however, that for autologous administration, new therapeutic approaches may be needed to restore MSC viability and function. Additional research is also required to characterize MSC viability and function across the spectrum of severity and presentations of preeclampsia.

## Perspectives and significance

Current results provide proof-of-concept evidence regarding the role of MSC senescence in the pathophysiology of preeclampsia. This may result in identifying new biomarkers and novel therapeutics using MSC and/or drugs that target fundamental senescence processes.

## Data Availability

The datasets used and/or analyzed during the current study are available from the corresponding author on reasonable request.
